# Early-Life Exposure to Formaldehyde through Clothing

**DOI:** 10.3390/toxics10070361

**Published:** 2022-06-30

**Authors:** Marta Herrero, Neus González, Joaquim Rovira, Montse Marquès, José L. Domingo, Martí Nadal

**Affiliations:** 1 Laboratory of Toxicology and Environmental Health, School of Medicine, Universitat Rovira i Virgili, 43201 Reus, Spain; marta.herrero@urv.cat (M.H.); nieves.gonzalez@urv.cat (N.G.); montserrat.marques@urv.cat (M.M.); joseluis.domingo@urv.cat (J.L.D.); marti.nadal@urv.cat (M.N.); 2 Institut d’Investigació Sanitària Pere Virgili (IISPV), 43204 Reus, Spain; 3 Environmental Engineering Laboratory, Departament d’Enginyeria Química, Universitat Rovira i Virgili, 43007 Tarragona, Spain

**Keywords:** formaldehyde, textiles, pregnant women, children, dermal absorption, risk assessment

## Abstract

Clothes contain a wide range of chemicals, some of them potentially hazardous. Recently, there has been a growing interest in eco-friendly clothing, including the use of organic cotton. However, the process of eco-friendly fabric production does not exclude the use of toxic substances, such as formaldehyde, a known human carcinogen. The present investigation was aimed at determining the presence of formaldehyde in eco-friendly and conventional clothing of pregnant women, babies, and toddlers from the Catalan (Spain) market. The potential effects of washing were also investigated by comparing the reduction of formaldehyde in unwashed and washed clothing. Formaldehyde was detected in 20% of samples, with a mean level of 8.96 mg/kg. Formaldehyde levels were surprisingly higher in eco-friendly than in regular garments (10.4 vs. 8.23 mg/kg). However, these differences were only significant (*p* < 0.05) for bras (11.6 vs. 7.46 mg/kg) and panties (27.1 vs. 6.38 mg/kg) of pregnant women. Dermal exposure and health risks were assessed for three vulnerable population groups: pregnant women, babies, and toddlers. In general, exposure was higher in babies (up to 1.11 × 10^−3^ mg/kg/day) than in other groups (2.58 × 10^−4^ and 4.50 × 10^−3^ mg/kg/day in pregnant women and toddlers, respectively). However, both non-carcinogenic and carcinogenic risks were below the safety limits (<1 and <10^−5^, respectively) according to national regulations. Notwithstanding, although formaldehyde levels were below the legal limits (<75 mg/kg) and health risks were within acceptable ranges, clothing may contain other toxic substances in addition to formaldehyde, thus increasing the risks. Finally, since no formaldehyde was detected in washed textile samples, a safe and simple practice for the consumers is to wash clothing before the first use.

## 1. Introduction

The impact of the textile industry on the environment and the human health is quite important. The large carbon footprint associated with its production, the elevated water consumption, the lack or low recycling rates, the huge amount of waste generated, and the use of chemical substances make the textile industry one of the most polluting productive sectors [1].

Clothes may contain a wide range of chemicals. Although a few substances result from the manufacturing, most chemicals are intentionally added. Skin is constantly in close contact with textiles. Therefore, these substances can be associated with skin adverse effects such as allergies, dermatitis, sensitisation, or reduction of microflora, among others [2,3,4]. The variety of substances used in the textile industry includes, among others, toxic metals [5,6,7], flame retardants [8], pesticides [9,10], dyes [6,11], and plasticisers [12]. Some of these substances are used to improve and make the manufacturing process more efficient [13]. Other substances are used to add new properties to fabrics, including antimicrobial activity [14], water repellence [15], flame retardancy [16], colour resistance, or breathability [17]. To date, research has mainly focused on the quantification of chemicals rather than their possible release from textiles. However, once substances are released from textiles, they can be absorbed through the skin, causing potential systemic and/or carcinogenic effects [6,18,19].

Eco-friendly production has rapidly become one of the new trends in the textile industry. New production policies involve a reduction of water and chemicals and the use of organic cotton in order to reduce the environmental impact. However, organic cotton refers only to cotton obtained through organic cultivation, but not to the addition of chemicals during the manufacture of the fibres [20]. Therefore, despite the use of more sustainable raw materials, textiles can accumulate chemicals from their local environment or during their manufacturing process, eventually posing health risks to consumers [21].

Textiles are products used by the entire population. Consequently, all consumers are exposed to their associated chemicals, although some population groups are more vulnerable than others. The early-life stages are critical for the further development of new-borns and infants, hence becoming vulnerable population groups.

Formaldehyde is one of the most widely used chemicals in the world. It is found in a variety of consumer products, such as furniture, glues, adhesives, insulation, paper coatings, disinfectants, tobacco, cosmetics, and textiles [22]. In the mid-1920s, it was introduced into the textile industry to increase the resistance of fabrics (e.g., cotton and polyester) to wrinkling during wear and washing [23]. Nowadays, it is also used as crosslinking, anti-mould, and as a dye-fixing agent [24,25,26], or for bleaching [27]. Nevertheless, like many other substances added to the fabrics, it is potentially toxic, and it can cause skin and eye irritation, as well as sensitization and toxic effects at the contact site [28]. In addition, formaldehyde is carcinogenic to humans according to the International Agency for Research on Cancer, with sufficient evidence of causing nasopharynx cancer, leukaemia, and sinonasal cancer [29,30]. The health risks of formaldehyde depend not only on its concentration, but also on the exposure route and time. The main risk is associated with inhalation, which may cause discomfort or nausea, stemming from the chemical’s pungent odour, irritation of the eyes, nose, and throat, and exacerbation of asthma [31,32]. Clothes are in direct contact with the skin and having formaldehyde in the clothing is associated with dermatitis, eczema, allergies, sensitization [31,33,34,35], and even increased cell proliferation in melanomas [36]. Unfortunately, although formaldehyde is toxic, nowadays it is still used in the textile industry. Specifically, it is used as a reducing agent during the dyeing step and in the finishing step (urea–formaldehyde resin) to reduce the formation of wrinkles in fabrics [37].

The washout effect during laundry has been studied for different chemicals, such as benzothiazoles [38] and titanium dioxide [39], as well as for microplastics [40]. In most cases, the contents of toxic substances significantly decrease after a few washings, leading to a reduced dermal exposure. However, the release of these pollutants ultimately means that they are a serious source of toxic substances and microplastics to the aquatic habitat [41]. Because of its high volatility, washing clothes may have an important effect on the contents of formaldehyde in clothing. Notwithstanding, it has not been largely proved in the scientific literature.

The present investigation was aimed at determining the presence of formaldehyde in clothes and its associated dermal exposure and human health risks, including “eco-friendly” labelled, used by pregnant women, babies, and toddlers from the Catalan (Spain) market.

## 2. Materials and Methods

### 2.1. Sampling

One hundred and twenty first-layer textile items usually worn by pregnant women, babies (<12 months old), or toddlers (12 to 36 months old) were purchased in hypermarkets, chain stores and small retailers of Tarragona County (Catalonia, Spain), and also at online shops. Once at the laboratory, all samples were wrapped in aluminium foil. The characteristics of each clothing item regarding type of fibre, colour, manufacture location, and density, are detailed as Supplementary Information (Table S1). For the three population groups, different clothing types were considered: elastic T-shirts, jeans/trousers/leggings, bras and panties for pregnant women; bodysuits, pyjamas, and socks for infants (<12 months old); and pyjamas, underwear, T-shirts, dresses, and trousers/leggings for toddlers (12–36 months old). Ten items of each one of these categories, covering different characteristics (i.e., colour, origin, material), were sampled. One-half of the samples were organic, either made of organic cotton or free of chemicals.

### 2.2. Determination of Formaldehyde

Free and extractable formaldehyde analysis was based on the 14184-1:2011 ISO norm. Approximately 2.5 g of sample was cut into small pieces (0.5 cm × 0.5 cm), inserted into an amber vessel with 100 mL of distilled water, and incubated in a water bath for 2.5 h at 40 °C with agitation. Afterwards, the extract was filtered and made up to a volume of 5 mL. Five mL of Nash reagent was then added, and the solution incubated in a water bath for 30 min at 40 °C with agitation. The Nash reagent was prepared by dissolving 150 g of ammonium acetate (≥98% Sigma-Aldrich, Darmstadt, Germany) in distilled water, adding 3.0 mL of glacial acetic acid (99.8%, Labkem, Spain) and 2.0 mL of acetylacetone (≥99% Sigma-Aldrich, Darmstadt, Germany), to a final volume of 1000 mL made up with distilled water. The solution was kept at room temperature for >12 h before its first use. The final extract was cooled at room temperature. The absorbance at 412 nm was read by spectrophotometry, using a UV/Vis Spectrophotometer (Cecil Aurius Series CE 2021, Cecil Instruments Limited, Cambridge, UK). Blanks and two replicates were also analysed every batch of 7 samples to assure the accuracy of the analytical method. The limit of detection was set as 3 times the signal-to-noise ratio set at a concentration of 12.8 mg/kg.

### 2.3. Washout Effect

Ten samples of jeans/trousers/leggings and 10 samples of panties of pregnant women were washed in a domestic washing machine, and the levels of formaldehyde before and after the washing were compared. The laundering was performed by using an LG F12C3QDP washer, using regular liquid detergent (Jabón de Marsella y Flor de Azahar, Carrefour, Spain; Composition: aqua, ethoxylated alcohol, dodecylbenzene sulphonic acid, sodium laureth sulfate, fatty acids, sodium tallowate, sodium cocoate, polyethyleneimine ethoxylated, sodium chloride, sodium hydroxide, parfum, polypropylene terephthalate, potassium sorbate, phenoxyethanol, modified phosphonate, modified phosphonate, stylbene derivative brightener, styrene/acrylate copolymer, dimethicone) and cleaning vinegar as softener. The clothes were subjected to a quick washing program for 30 min at cold temperature and 1200 rpm, and in the open air. The chemical analyses of formaldehyde were conducted using the same UV/Vis spectrophometer as for unwashed samples.

### 2.4. Exposure Assessment and Risk Characterization

The concentration of free and extractable formaldehyde in clothes was used to calculate the irritation and sensitization, considering each garment individually. Both were determined by dividing the formaldehyde concentration extracted from the garment by no-observed-adverse effect concentrations (NOAEC) of 0.005% (*w*/*w*) [42].

In addition, dermal exposure of formaldehyde through contact textiles was estimated for three different groups: pregnant women, infants (<12 months old), and toddlers (boys and girls, 12–36 months old). A conservative scenario (use of long sleeve tops and long trousers) was considered for calculations, which were based on the following equation, developed by the European Chemical Agency (ECHA) [43].

$${\mathrm{Exp}}_{\mathrm{derm}}=\frac{F_{\mathrm{cloth}}{\;\times \;d}_{\mathrm{cloth}}{\;\times \;A}_{\mathrm{skin}}{\;\times \;F}_{\mathrm{mig}}{\;\times \;F}_{\mathrm{contact}}{\;\times \;F}_{\mathrm{pen}}{\;\times \;T}_{\mathrm{contact}}\;\times \;n}{\mathrm{BW}}\;$$

where Exp_derm_ is the dermal exposure (mg/(kg·day)), F_cloth_ is the fraction of element in clothes (dimensionless), d_cloth_ is the density of the clothing (mg/cm^2^); A_skin_ is the skin area covered by the clothing (cm^2^), F_mig_ (%) is the migration fraction of substance from cloth to skin, F_contact_ is the fraction of contact area for skin (dimensionless) [44], F_pen_ is the penetration rate of the element (dimensionless), T_contact_ is the duration of the clothing skin contact (day), n is the number of events per day (1/day), and BW is the body weight (kg). The dermal exposure parameters are summarized in Table 1.

Non-carcinogenic risks were determined by calculating the hazard quotient (HQ), which is defined as the quotient between exposure and the dermal reference dose (RfD). In turn, the carcinogenic risk was calculated multiplying the exposure by the respective dermal slope factor (SF). RfDs and SFs were obtained from the Regional Screening Level from the U.S. EPA Preliminary Remediation Goals [45].

Since formaldehyde is a very volatile compound, the health risk through air inhalation was also evaluated. According to previous studies, inhalation can be considered as the main exposure pathway to this compound, as well as other volatile organic compounds (VOCs) [49], especially in indoor environments [50]. Formaldehyde concentrations in air were obtained from Rovira et al. [51], who analysed the indoor concentration of this chemical in homes of Catalonia. Inhalation exposure levels (Exp_inh_) were calculated according to the following equation:

$${\mathrm{Exp}}_{\mathrm{inh}}=\frac{{(C}_{i}{\;\times \;\mathrm{IR}}_{i}{\;\times \;F}_{i})\;\times \;\mathrm{EF}}{\mathrm{BW}\;\times \;365}$$

where C_i_ is the concentration of formaldehyde in air (in µg/m^3^), IR_i_ is the inhalation rate (in m^3^/day), F_i_ is the daytime fraction spent indoors (unitless), EF is the exposure frequency (in day/year), BW is the body weight (in kg), and 365 is a conversion unit factor (in day/year). Non-carcinogenic risks associated to formaldehyde inhalation were calculated as the relationship between exposure and the inhalation reference dose (RfD_inh_). On the other hand, carcinogenic risks were also assessed by multiplying the predicted exposure concentration by the inhalation unit risk (IUR). The inhalation exposure parameters are shown in Table 2.

### 2.5. Statistics

Data analysis was carried out using the IBM SPSS Statistics software version 27.0 (IBM Corp. Released 2020, Armonk, NY, USA). The Kolmogorov–Smirnov test was used to assess the distribution of the values. In turn, Student’s t-test or ANOVA test for data following a parametric distribution, or the Kruskal–Wallis tests for non-parametric data were used to assess differences between groups. A difference was considered statistically significant when the probability was lower than 0.05 (*p* < 0.05). Non-detected (ND) levels were considered as one-half of the detection limit (DL) (ND = 1/2DL).

## 3. Results

### 3.1. Analysis of Formaldehyde

The concentrations of free and extractable formaldehyde in the clothing of different population groups are shown in Table 3.

Formaldehyde was detected in 20% of the samples, with a mean level of 8.96 mg/kg (range: <12.8 to 55.7 mg/kg). Two pregnancy panties and a sample of baby socks were the garments with the highest burdens of formaldehyde (55.7, 37.3, and 24.5 mg/kg, respectively).

**Figure 1:**
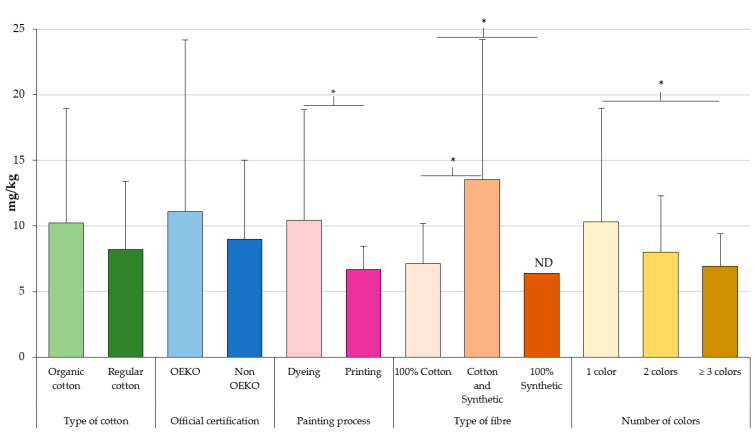
Formaldehyde concentrations according to a number of factors, including cotton production, OEKO-TEX^®^ Standard 100, painting process, type of fibre, and number of colours. * An asterisk indicates significant differences at *p* < 0.05. ND: Not detected.

The mean formaldehyde content of each fabric according to several factors (e.g., organic cotton, type of fibre, OEKO certification, dying/painting, number of colours) is depicted in Figure 1. Dyed garments showed significantly (*p* < 0.05) higher levels than printed ones (10.2 vs. 7.07 mg/kg). Considering the type of fibre material, 100% cotton, 100% synthetic, and a combination of both, formaldehyde was detected in 22%, 14%, and 47% of the samples, respectively, proving that synthetic clothes have lower contents of formaldehyde. The garments made of a mixture of cotton and synthetic fibres (12.7 mg/kg) had significant (*p* < 0.05) higher levels than those made of 100% cotton (7.51 mg/kg) or 100% synthetic fibres (6.66 mg/kg). In turn, monochromatic clothing (10.1 mg/kg) had significantly (*p* < 0.05) higher concentrations than garments, with two (8.33 mg/kg) and more than two colours (7.29 mg/kg). Formaldehyde concentrations were also higher in OEKO Standard 100 garments than non-OEKO certified, but this difference was not significant (*p* > 0.05).

Up to 40 items of pregnant women’s clothing, including T-shirts, trousers/jeans/leggings, bras and panties, were analysed. Among them, trousers and underwear were the items with the highest mean concentrations (18.2 and 12.5 mg/kg, respectively). Particularly, the sample with the greatest amount was #17, corresponding to grey jeans made of 86% cotton (20% recycled cotton from post-consumer textile waste from collected garments) and 12% polyester (Supplementary Information, Table S1), with a value of 24.5 mg/kg. Two black panties (sample #31 and sample #34, showed the highest formaldehyde values of the study (37.3 mg/kg and 55.7 mg/kg), both made of 100% organic cotton.

Pyjamas, bodysuits, and socks are undoubtedly the items of baby clothing with the most direct and prolonged contact with the skin. Ten samples from each category were analysed, formaldehyde residues being detected only in a few socks, sample #63 (24.5 mg/kg), both made of a combination of polyamide, cotton, and elastane.

With respect to toddlers’ clothing, pyjamas, underwear, dresses, T-shirts, leggings, and jeans were analysed. Up to 40% of the samples of trousers/jeans, one-half of them made of organic cotton (*n* = 2), presented detectable concentrations of formaldehyde, with levels ranging between 13.1 and 16.4 mg/kg. In turn, only 10% of the T-shirts and dresses (*n* = 2, made of organic cotton) showed traces of formaldehyde. Finally, no traces of formaldehyde were found in the samples of pyjamas and underwear.

**Figure 2:**
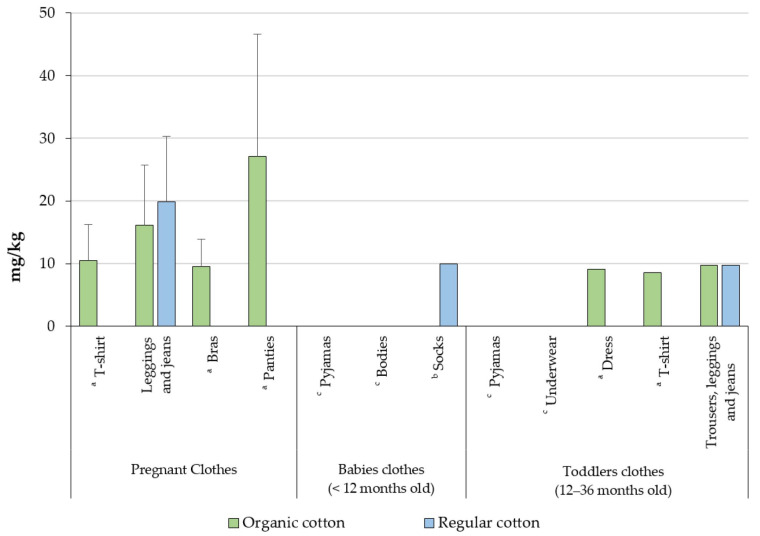
Formaldehyde concentrations according to type of cotton in clothes for pregnant women, babies aged <12 months, and toddlers aged 12–36 months. ^a^ Not detected in regular cotton. ^b^ Not detected in organic cotton. ^c^ Not detected in any types of cotton.

T-shirts, bras, and panties of pregnant women, as well as T-shirts and dresses of toddlers, made from organic cotton, showed higher levels of formaldehyde than those made from conventional materials (Figure 2).

Only 25 samples had an official certification. Twelve of them were certified with the OEKO-TEX^®^ Standard 100 label, while two were OEKO-TEX^®^ Made in Green, and 11 had GOTS certification. The regulation of the OEKO-TEX^®^ Standard 100 and OEKO-TEX^®^ Made in Green labels states a maximum residue level of 75 mg/kg for formaldehyde, which was achieved by all the certified garments. In turn, the GOTS certification is the most restrictive regulation, as certified textile samples must be free of formaldehyde. Despite this, a quantifiable amount of this chemical (20.0 mg/kg) was found in sample #92 (GOTS certified dark blue dress, 100% organic cotton).

The effect of washing clothes, in terms of releasing formaldehyde, was evaluated in some garments with the greatest concentrations of this substance (10 samples of jeans/trousers/leggings and 10 samples of panties, all of them for pregnant women). For that purpose, formaldehyde levels in unwashed and washed samples were compared. None of the 20 clothing items showed detectable amounts of formaldehyde after washing (<12.8 mg/kg).

### 3.2. Human Exposure and Risk Assessment

The dermal exposure of formaldehyde, based on the mean concentration of each garment, was assessed for the same population groups: pregnant women, babies of <12 months, and toddlers aged 12–36 months. The individual exposure, considering each textile category, and the total exposure, considering that people wear several clothes at the same time, are summarised in Table 4. Baby socks were identified as the category with the highest exposure (5.13 × 10^−4^ mg/kg/day), followed by trousers/leggings/jeans (3.30 × 10^−4^ mg/kg/day). In general terms, total exposure was 2-times higher in babies (1.11 × 10^−3^ mg/kg/day) than in toddlers (4.50 × 10^−4^ mg/kg/day), while the lowest exposure to formaldehyde corresponded to pregnant women.

Dermatitis or local allergic reactions are probably the most common adverse effects of short-term dermal exposure to formaldehyde [29,34,43]. In the current study, the risk of sensitisation was calculated by using the content of formaldehyde in clothing. Formaldehyde extracted from all garments showed values at least 10 times lower than NOAEC (0.005% *w*/*w*), being 0.1% the value at which signs of sensitisation could be observed [42].

**Figure 3:**
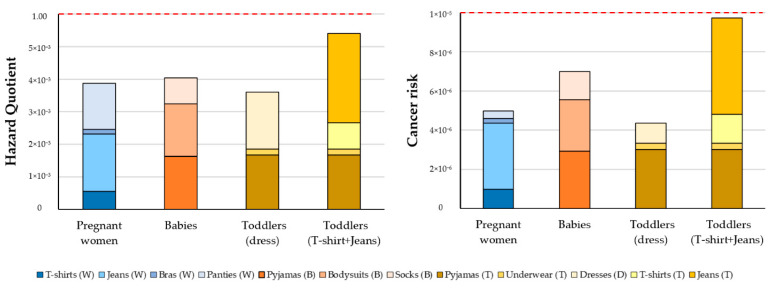
Risk assessment of dermal exposure to formaldehyde in the worst-case scenario, for pregnant women (W), babies (B) aged <12 months, and toddlers (T) aged 12–36 months.

The hazardous quotient (HQ), estimated from the dermal exposure and the RfD, was far below the limit value, which is set to unity. It is a clear indication that formaldehyde in clothing does not currently mean non-cancer risks for the population. On the other hand, cancer risks ranged from 1.38 × 10^−7^ to 9.49 × 10^−7^ for pregnant women, from 4.61 × 10^−7^ to 2.76 × 10^−7^ for babies and from 8.13 × 10^−7^ to 2.97 × 10^−6^ for toddlers. All values are below the limit of 10^−5^, the Spanish threshold level. Non-cancer and cancer risks of dermal contact to formaldehyde are shown in Figure 3.

Dermal absorption is not the only pathway of exposure to formaldehyde. Air inhalation is considered as the most serious exposure pathway for this chemical. In 2014, Rovira et al. [51] conducted a study in which formaldehyde air levels were analysed in different spaces, homes, and workplaces (shops, offices, schools). When analysing the total exposure to formaldehyde, the contribution of the inhalation route was found to be much higher than the dermal route, with a percentage of 90% of the total. Therefore, it is evident that clothing is not the most relevant exposure pathway to formaldehyde.

## 4. Discussion

Because formaldehyde is a usual chemical additive in clothing, its presence in textile materials has been largely studied, especially when assessing the compliance of some certifications, such as OEKO-TEX^®^ Standard 100. However, information on the benefits of using organic cotton before conventional raw materials is scarce. Furthermore, there is a lack of investigations aimed at evaluating the contribution of dermal exposure to formaldehyde through clothing in front of other exposure pathways, such as air inhalation.

Results of previous studies performed worldwide on the contents of formaldehyde in clothes are summarized in Table 5. There is a wide variability in the reported values, possibly due to the use of different analytical methodologies and/or commercial regulations. In 2010, the U.S. Government Accountability Office conducted a study on a representative sample of 180 items. Ten of them exceeded the threshold value of 75 mg/kg, with a maximum of 206.1 mg/kg [30]. In 2018, Caro Zapata et al. [53] found formaldehyde in 74% of the analysed samples in Colombia, reporting a maximum concentration of 87 mg/kg. These levels were similar to those found by Aldag et al. [23], who reported a maximum level 75.9 mg/kg in several T-shirts and pants purchased in Germany. By contrast, formaldehyde levels in 34 textile samples acquired in South Africa were found within the range 0.036–0.090 mg/kg [54].

Focusing on Europe, there is a clear tendency towards a reduction in detection rates and concentrations. In 2007, a study in the European Union quantified the formaldehyde present in different types of textiles, stating that 10% of all samples released more than 30 mg/kg formaldehyde, with 3% even exceeding the limit of 75 mg/kg [58]. In 2017, Aldag et al. [23] carried out a new study that also included curtains, in addition to clothing. The maximum concentrations were substantially lowered from 166 to 80 mg/kg [23]. Our results confirm this decreasing trend.

In recent years, there has been an increasing awareness of eco-friendly fashion with the use of more sustainable textile materials, such as organic cotton. In order to determine whether eco-friendly clothing contains fewer chemical additives, 50% of the garments under study were organic. Surprisingly, in 5 out of the 12 categories, organic cotton clothes showed higher levels of formaldehyde than clothing made of regular cotton. It must be highlighted that regulations of using organic cotton in textile manufacturing refer only to pesticide-free cultivation, but not to the addition or accumulation of toxic substances in clothing [20]. The current results agree with those of other studies, focused on other chemicals [59]. In a previous investigation, significantly higher copper values were found in eco-labelled jeans than in conventional items [6]. However, it should be taken into account that the garments here analysed are brand new. Formaldehyde levels are expected to decrease with exposure to light, and as shown, even a single wash would be sufficient to reduce these concentrations.

By labelling garments, the textile industry informs consumers about the origin of the products, and how taking care of the garment to reduce its environmental impact. The most commonly used terms are “environmentally friendly”, “nature-friendly”, “ethical”, “green”, “responsible”, “ecologically clean”, “ecologically innocuous”, “eco-conscious”, “eco-friendly”, “organic” and “sustainable” [60]. In the present study, samples of the last four were collected. In Europe, there are three voluntary labelling systems for textiles: the European Eco-label [61], the OEKO-TEX^®^ Standard 100 label, and Global Organic Standard Textiles [62], which is the most restrictive. In the European Union, the current limit for formaldehyde in clothing in contact with the skin is 75 mg/kg [63], which is the same threshold set for OEKO-TEX^®^ Standard 100 label. On the other hand, the Eco-label certification establishes a limit of 30 mg/kg, and OEKO-TEX^®^ Standard 100 label sets 75 mg/kg. Moreover, textiles for babies are subjected to more restrictive limits, as they should release less than 20 mg/kg of formaldehyde [64]. Finally, the limit according to the GOTS certification is 16 mg/kg in all the textiles [62]. Despite the value that these labels bring to garments, their use is not yet widespread. In this study, only 21% of the garments had a label of this type. Exceptionally, one of the samples, GOTS certified, did not achieve the specifications in terms of formaldehyde content.

The changes in textile production over the past years, towards an eco-friendlier production with the environment, remain unprecedented. These new production policies are focused on minimising the environmental impact, from production to the life of the clothing [65]. These practices include reduction of water consumption, decrease of waste generation, and recycling of garments, among others. In addition, producers tend to reduce the use of chemicals that are hazardous to both the environment and human health [66,67]. Some studies have shown that eco-garments may contain lower levels of some trace elements, such as aluminium and strontium, than non-eco garments [18]. The concentrations here reported were lower than those reported in the 2007 European survey on the release of formaldehyde from textiles [55], where 11% of samples intended to be in direct contact with skin exceeded 30 mg/kg, and even 3% overpassed the threshold of 75 mg/kg [55]. This decrease could be a consequence of the effectiveness of the new sustainability policies of the textile industry and the commitment of brands to make safer garments for consumers.

Exposure to pollutants does not occur by a single route, but people are exposed to chemicals through different pathways [58]. Food ingestion plays a relevant role, but inhalation, ingestion, and dermal absorption may be also quite contributory. In fact, air inhalation has been identified as the most important exposure pathway for formaldehyde and other VOCs. Formaldehyde is a known indoor pollutant present in many household items. Wood-pressed products, insulation materials, paints, varnishes, household cleaning products, cigarettes, and candle burning, among others, are the main sources of this indoor pollutant [66]. Therefore, most formaldehyde risk assessment studies have focused on the estimation of inhalation risks [51,68,69,70]. Here, we demonstrated that dermal absorption of formaldehyde contained in clothing is less important than air inhalation (10% vs. 90%).

Washing clothes may be a significant way to reduce the content of formaldehyde and other textile additives. The washout effect is very relevant, as washed samples contained no traces of formaldehyde after only one washing, irrespective of the concentrations in unwashed samples. This is critical information for public health authorities, whose recommendation, especially for early-life population groups, should be to wash all the clothing before the first use. This simple practice is an effective way to substantially reduce the amount of formaldehyde in garments and, consequently, to decrease any potential health risks.

## 5. Conclusions

Formaldehyde was detected in 20% of the samples analysed with levels ranging from <12.8 mg/kg to 55.7 mg/kg. Formaldehyde levels were below the EU limits (<75 mg/kg) in all the textile samples. The levels in the dyed garments were higher, 10.2 mg/kg, than those found in the printed garments, 7.07 mg/kg. Regarding the material from which the garments are made, clothing made from cotton contained higher concentrations of formaldehyde than clothing made from synthetic fibres (7.51 mg/kg vs. 6.66 mg/kg). Interestingly, eco-friendly clothes contained traces of formaldehyde. In some cases (e.g., underwear of pregnant women, dresses and T-shirts of toddlers) even at higher levels than conventional garments. In spite of this, the dermal contact to formaldehyde through clothing was not significant, being estimated as almost 10-times lower than the air inhalation of formaldehyde, which is the most relevant exposure route to this chemical. Furthermore, both non-carcinogenic and carcinogenic risks were below the safety limits. A simple but very effective practice to reduce these risks would be to wash all the textile items before the first use. Clothing may contain other toxic substances apart from formaldehyde. Therefore, future health risk assessments should be performed under a multi-exposure and multi-chemical scenario, considering also individual susceptibility.

## Figures and Tables

**Table 1:** Parameters used to assess dermal exposure.

Variable	Description	Value	Reference
F_cloth_	Weight fraction of substance in garments	Cloth specific mg/mg	Supplementary Table S1
d_cloth_	Clothing grammage	Cloth specific mg/cm^2^	Supplementary Table S1
A_skin_	Pregnancy women t-shirt (trunk + arms)	8910 cm^2^	[46]
	Pregnancy Troussers_Jeans_Leggings (legs)	5980 cm^2^	
	Pregnancy Band of Trouser (trunk/2)	3270 cm^2^	
	Pregnancy Troussers_Jeans_Leggings + Band (legs + trunk/2)	9250 cm^2^	
	Pregnancy Bra (Bosom)	2594 cm^2^	
	Pregnancy underwear without brand (genitals and buttocks)	1469 cm^2^	
	Pregnancy underwear with brand (genitals and buttocks +trunk/2)	4739 cm^2^	
	Baby Pyjamas (Trunk + Arms + Legs + Feet)	2778 cm^2^	
	Baby Bodysuits (trunk + arms)	1795 cm^2^	
	Baby socks (Feet)	235 cm^2^	
	Toddlers Pyjamas (Trunk + arms + legs)	4355 cm^2^	
	Underwear (Genitals)	383 cm^2^	
	Dresses (Trunk + arms + 1/2legs)	3665 cm^2^	
	T-shirt (trunk + arms)	2975 cm^2^	
	Trousser/Jeans/leggings (Legs)	5980 cm^2^	
F_contact_	Fraction of contact area for skin	1	[44]
F_mig_	Migration fraction from cloth to skin	0.5%	[44]
F_pen_	Fraction of penetration inside the body	0.01	[42]
T_contact_	Contact duration between skin-textile	0.33 (8 h/24 h) 0.67 (16 h/24 h) 1 (24 h)	Assumed
N	Mean number of events per day	1/d	Assumed
BW	Adult Female	76.9 kg	[47]
	Birth to <12 month	7.31 kg	[48]
	1 < 3 years	12.5 kg	

**Table 2:** Parameters used to assess inhalation exposure.

Variable	Description	Value	Reference
C_i_	Air concentration		
	Bedroom	27.3 µg/m^3^	[51]
	Living room	22.5 µg/m^3^	
	Outdoor	1.62 µg/m^3^	
	Work	21.8 µg/m^3^	
IR_i_	Inhalation rates		
	Pregnant women	19.2 m^3^/day	[52]
	Infants <12 months	5.40 m^3^/day	
	Toddlers 12–36 months	8.45 m^3^/day	
F_i_	Time fraction		
	Bedroom	0.36	[51]
	Indoor (excl. bedroom)	0.37	
	Outdoor	0.10	
	At work	0.14	
EF	Exposure frequency	350 days/year	[51]
BW	Body weight		
	Pregnant women	76.9 kg	[47]
	Infants <12 months	7.31 kg	[48]
	Toddlers 12–36 months	12.5 kg	

**Table 3:** Free and extractable formaldehyde levels (mg/kg) in the clothing of pregnant women, babies and toddlers purchased from Catalonia (Spain).

		Detection Rate (%)	Mean	SD	Minimum	Maximum
Pregnant women’s clothes	T-shirts (*n* = 10)	20	8.44	4.42	<12.8	18.4
	Jeans/leggings (*n* = 10)	80	18.2	9.77	<12.8	24.5
	Bras (*n* = 10)	20	7.94	3.35	<12.8	15.5
	Panties (*n* = 10)	40	16.7	17.0	<12.8	55.7
Babies clothes (<12 months)	Pyjamas (*n* = 10)	0	<12.8	0.00	<12.8	<12.8
	Bodysuits (*n* = 10)	0	<12.8	0.00	<12.8	<12.8
	Socks (*n* = 10)	10	8.19	5.72	<12.8	24.5
Toddlers clothes (12–36 months)	Pyjamas (*n* = 10)	0	<12.8	0.00	<12.8	<12.8
	Underwear (*n* = 10)	0	<12.8	0.00	<12.8	<12.8
	Dresses (*n* = 10)	10	7.74	4.29	<12.8	20.0
	T-shirts (*n* = 10)	10	7.48	3.49	<12.8	17.4
	Trousers (*n* = 10)	40	9.87	4.65	<12.8	16.4

SD: Standard deviation.

**Table 4:** Dermal exposure (mg/kg/day) to formaldehyde through clothing.

		Dermal Exposure per Item	Total Exposure (Non-Cancer Risk)
Pregnant women	T-shirts	5.23 × 10^−5^	2.58 × 10^−4^
	Jeans/leggings	1.48 × 10^−4^	
	Bras	1.54 × 10^−5^	
	Panties	4.23 × 10^−5^	
Babies (<12 months old)	Pyjamas	3.07 × 10^−4^	1.11 × 10^−3^
	Bodysuits	2.92 × 10^−4^	
	Socks	5.13 × 10^−4^	
Toddlers (12–36 months old)	Pyjamas	2.68 × 10^−4^	4.50 × 10^−4^ * 1.44 × 10^−4^ **
	Underwear	2.99 × 10^−5^	
	Dresses	1.14 × 10^−4^	
	T-shirts	8.96 × 10^−5^	
	Trousers	3.30 × 10^−4^	

* Dressed with underwear, T-shirt and trousers. ** Dressed with underwear and dress.

**Table 5:** Evolution of the concentration of formaldehyde in clothes.

Country	Year	Number and Type of Clothes	Detection Rate	Formaldehyde Content	Reference
Spain	2022	124 samples	19%	ND–56 mg/kg	This study
South Africa	2019	34 socks samples	0%	100% ND	[54]
Colombia	2018	62 samples	74%	ND–87 mg/kg	[53]
Europe	2017	4 curtains, 4 pants, 14 T-shirts and 2 shirts	71%	ND–76 mg/kg	[23]
European Union	2007	221 samples (48 water extraction)	48%	ND in 52%; 30–166 mg/kg in 48%	[55]
Denmark	2003	10 textiles	30%	ND in 70%; 35–82 mg/kg in 30%	[56]
USA	1998	16 fabrics	50%	ND in 50%; <200 p.p.m in 50%	[57]

ND: not detected.

## Data Availability

No applicable.

## Supplementary Materials

The following supporting information can be downloaded at: https://www.mdpi.com/article/10.3390/toxics10070361/s1, Table S1: Main characteristics of the clothes analysed.
