# Supplementary material for: Early-Life Exposure to Formaldehyde through Clothing
Source: Toxics. 2022 Jun 30;10(7):361. doi: 10.3390/toxics10070361 (PMC9318620; doi:10.3390/toxics10070361)
Supplement: Supplementary file 1 [file toxics-10-00361-s001.zip › toxics-1773791-supplementary.pdf]

Table S1. Main characteristics of the clothes analysed.

No.		Type of clothes	Place of purchase	Materials	Regular Cotton	Made in	Colour	Official certification	Density	Comments
1		T-shirt	Chain Store	95% C, 5% E	*	Bangladesh	White		16.0	
2		T-shirt	Chain Store	95% C, 5% E	*	Bangladesh	Black		15.6	
3		T-shirt	Chain Store	95% C, 5% E	*	Bangladesh	Beige		15.1	
4		T-shirt	Chain Store	95% C, 5% E	*	Bangladesh	Black and white		14.7	
5		T-shirt	Chain Store	95% C, 5% E	*	Bangladesh	Pink		20.5	
6		T-shirt	Supermarket	100% C		Bangladesh	Blue and white	OEKO-TEX® Standard 100	14.6	
7		T-shirt	Supermarket	95% C, 5% E		Bangladesh	Mustard and white	OEKO-TEX® Made in Green	16.0	
8		T-shirt	Supermarket	95% C, 5% E		Bangladesh	Dark Blue	OEKO-TEX® Made in Green	16.6	
9		T-shirt	Chain Store	95% C, 5% E		Pakistan	Yellow		18.2	
10		T-shirt	Chain Store	95% C, 5% E		Bangladesh	Blue and stamp		18.1	
11a		Rib	Chain Store	95% C, 5% E	*	Bangladesh	Grey		20.4	
11b		Jogger	Chain Store	65% P, 35% V		Bangladesh	Grey		19.5	
12a		Rib	Chain Store	95% C, 5% E		Pakistan	Blue		19.5	
12b		Jean	Chain Store	71% C, 27% P, 3% E		Pakistan	Blue		19.0	
13a		Jean	Supermarket	98% C, 2% E		Pakistan	Blue		23.8	
13b		Rib	Supermarket	96% C, 4% E		Pakistan	Blue		38.1	
14	Pregnant women Clothes	Legging	Chain Store	92% C, 8% E		Sri Lanka	Black		27.6	
15		Trouser	Chain Store	62% P, 31% V, 7% E		Turkey	Cream		24.3	
16		Legging	Supermarket	95% C, 5% E	*	Bangladesh	Navy Blue	OEKO-TEX® Standard 100	16.5	
17a		Rib	Chain Store	95% C, 5% E	*	Bangladesh	Black		21.5	20% recycled
17b		Jean	Chain Store	86% C, 12% P		Bangladesh	Grey		37.9	
18		Legging	Chain Store	94% V, 6% E	*	Turkey	Black		23.1	
19		Legging	Chain Store	96% C, 4% E	*	India	Blue		18.1	
20		Jean	Chain Store	99% C, 1% E	*	Cambodia	Blue		42.9	
21		Bra	Chain Store	100% P		China	Black		24.4	Lining
22		Bra	Chain Store	100% P		China	Grey		27.7	Lining
23		Bra	Chain Store	95% C, 5% E		China	White		12.2	
24		Bra	Chain Store	95% C, 5% E		China	Grey		16.2	
25		Bra	Chain Store	90% C, 10% E		China	Grey and flowers		19.3	
26		Bra	Chain Store	95% C, 5% E	*	Bangladesh	Grey		14.1	Lining
27		Bra	Chain Store	95% C, 5% E	*	Bangladesh	White		11.7	Lining
28		Bra	Chain Store	95% C, 5% E	*	China	Grey	OEKO-TEX® Standard 100	15.2	
29		Bra	Chain Store	95% C, 5% E	*	Myanmar	Grey		17.0	
30		Bra	Chain Store	95% C, 5% E	*	Myanmar	White		14.4	
31		Panties	Chain Store	95% C, 5% E	*	Bangladesh	Black		16.1	

32		Panties	Chain Store	95% C, 5% E	*	Bangladesh	Pink		15.6
33		Panties	Chain Store	95% C, 5% E	*	Bangladesh	Grey		16.3
34		Panties	Chain Store	79% C, 15% PA, 6% E	*	Turkey	Black	OEKO-TEX® Standard 100	25.0
35		Panties	Chain Store	95% C, 5% E	*	China	Grey		17.3
36		Panties	Chain Store	95% C, 5% E		Turkey	Grey		16.1
37		Panties	Chain Store	95% C, 5% E		Turkey	Black		18.2
38		Panties	Chain Store	86% PA, 14% E		Italy	White		14.5
39		Panties	Chain Store	86% PA, 14% E		Italy	Black		14.5
40		Panties	Chain Store	95% C, 5% E		China	Grey		19.2
41		Pyjamas	Chain Store	100% C	*	Bangladesh	Beige and stamp		18.8
42		Pyjamas	Chain Store	100% C	*	Bangladesh	Pink and white		19.0
43		Pyjamas	Chain Store	100% C	*	Bangladesh	Pink		20.0
44		Pyjamas	Supermarket	100% C	*	India	Stamp white and grey	OEKO-TEX® Standard 100	18.7
45		Pyjamas	Shop	100% C	*	Turkey	White and green	GOTS*	19.9
46		Pyjamas	Supermarket	100% C		China	Pink and black		18.9
47		Pyjamas	Chain Store	100% C		Sri Lanka	Mustard and white	OEKO-TEX® Standard 100	17.9
48		Pyjamas	Chain Store	100% C		Sri Lanka	Beige	OEKO-TEX® Standard 100	18.3
49		Pyjamas	Chain Store	100% C		Sri Lanka	Stamp Green	OEKO-TEX® Standard 100	18.7
50		Pyjamas	Chain Store	100% C		China	White, green and yellow		18.5
51		Bodysuit	Chain Store	100% C	*	China	White		18.9
52		Bodysuit	Chain Store	100% C		Bangladesh	Beige and brown		19.1
53		Bodysuit	Chain Store	100% C		Bangladesh	Mustard and brown		18.6
54	Babies clothes (<12 months old)	Bodysuit	Chain Store	100% C	*	Cambodia	Green		19.3
55		Bodysuit	Chain Store	100% C	*	Cambodia	White		19.5
56		Bodysuit	Chain Store	100% C	*	Cambodia	Grey		20.6
57		Bodysuit	Chain Store	100% C		Bangladesh	Pink and white	OEKO-TEX® Standard 100	16.1
58		Bodysuit	Chain Store	100% C		Bangladesh	Pink	OEKO-TEX® Standard 100	18.5
59		Bodysuit	Chain Store	100% C		Bangladesh	White	OEKO-TEX® Standard 100	18.0
60		Bodysuit	Shop	100% C	*	EU	White, black and yellow	GOTS*	17.7
61		Socks	Chain Store	63% C, 33 PA, 4 E		China	White, camel and blue		31.4
62		Socks	Chain Store	75% C, 23% PA, 2% E		Pakistan	Grey and pink		30.4
63		Socks	Chain Store	78% C, 20% PA, 2% E		China	Violet		30.1
64		Socks	Chain Store	72% C, 26% P, 2% E		China	Dark grey		28.1
65		Socks	Supermarket	80% C, 20% PA		Barcelona	Dark blue		26.0

66		Socks	Shop	98% C, 2% E	*	Deutschland	Red and white		27.3	
67		Socks	Shop	98% C, 2% E	*	Deutschland	Blue and white		19.7	
68		Socks	Chain Store	76% C, 22% PA, 2% E	*	Turkey	Light Brown		29.4	
69		Socks	Chain Store	76% C, 22% PA, 2% E	*	Turkey	Cream		32.0	
70		Socks	Chain Store	76% C, 22% PA, 2% E	*	Turkey	White		33.2	
71		Pyjamas	Chain Store	100% C	*	China	Green		18.0	
72		Pyjamas	Supermarket	100% C	*	Bangladesh	Red	OEKO-TEX® Standard 100	17.6	
73		Pyjamas	Chain Store	100% C	*	Bangladesh	Green		22.4	
74		Pyjamas	Chain Store	100% C	*	Bangladesh	Mustard and white		19.5	
75		Pyjamas	Shop	100% C	*	Deutschland	Blue and white		19.0	
76		Pyjamas	Chain Store	100% C		India	White, red and yellow		19.8	
77		Pyjamas	Chain Store	100% C		India	Fluor		14.7	
78		Pyjamas	Chain Store	100% C		India	Black		15.7	
79		Pyjamas	Chain Store	100% C		Bangladesh	Blue and green		19.1	
80		Pyjamas	Supermarket	100% C		India	Blue and black		13.4	
81		Underwear	Supermarket	100% C	*	EU	White		18.4	
82		Underwear	Shop	100% C	*	Deutschland	White		17.8	
83		Underwear	Shop	100% C	*	Deutschland	Dark blue		17.9	
84		Underwear	Shop	100% C	*	Deutschland	Pink		18.4	
85	Toddlers clothes	Underwear	Shop	100% C	*	Deutschland	Pink		14.1	
86	(12-36 months	Underwear	Chain Store	100% C		India	Yellow		13.0	
87	old)	Underwear	Chain Store	100% C		India	White		13.2	
88		Underwear	Chain Store	100% C		Bangladesh	Rosa		13.0	
89		Underwear	Chain Store	100% C		Bangladesh	Green		12.6	
90		Underwear	Chain Store	100% C		China	Red		13.9	
91		Dress	Chain Store	100% C	*	Bangladesh	White and blue		15.1	
92		Dress	Shop	100% C	*	Deutschland	Dark blue		18.1	
93		Dress	Chain Store	100% C	*	Bangladesh	White and blue		14.4	
94		Dress	Shop	100% C	*	India	Stamp		18.6	
95		Dress	Shop	100% C	*	India	Stamp		14.1	
96		Dress	Chain Store	100% P		China	White		5.07	Lining
97		Dress	Chain Store	100% C		Bangladesh	Pink		15.4	
98		Dress	Chain Store	100% C		Bangladesh	Pink, green and white		16.6	
99		Dress	Chain Store	69% C, 28% P, 3% E		Portugal	Pink		22.5	
100		Dress	Supermarket	100% C		India	Blue and pink		5.44	Lining
101		T-shirt	Chain Store	100% C	*	Bangladesh	Pink		14.1	

102	T-shirt	Shop	100% C	*	Deutschland	Pink	16.8
103	T-shirt	Chain Store	100% C	*	Bangladesh	Blue and white	15.0
104	T-shirt	Chain Store	100% C	*	Turkey	Blue and red	14.6
105	T-shirt	Chain Store	100% C	*	Bangladesh	Green and white	14.6
106	T-shirt	Chain Store	100% C		Spain	Violet	18.8
107	T-shirt	Chain Store	100% C		Portugal	Grey	17.6
108	T-shirt	Chain Store	100% C		India	Pink	15.9
109	T-shirt	Chain Store	100% C		India	Cream, pink and blue	17.1
110	T-shirt	Supermarket	100% C		Bangladesh	White	16.0
111	Legging	Chain Store	95% C, 5% E	*	Bangladesh	White and black	17.5
112	Legging	Chain Store	95% C, 5% E	*	Bangladesh	Blue	18.7
113	Jean	Chain Store	100% C	*	Bangladesh	Blue	25.1
114	Legging	Chain Store	100% C	*	Bangladesh	Dark grey	17.4
115	Jean	Chain Store	100% C	*	Bangladesh	Black	22.7
116	Jean	Chain Store	98% C, 2% E		Morocco	Blue	34.4
117	Legging	Chain Store	95% C, 5% E		India	Green and Pink	18.7
118	Trouser	Supermarket	98% C, 2% E		Bangladesh	Camel	23.6
119	Trouser	Chain Store	69% C, 28% P, 3% E		Bangladesh	Brown	16.9
120	Trouser	Chain Store	100% C		Bangladesh	Green	26.2
